# SBA‐15 Supported Ni‐Cu Catalysts for Hydrodeoxygenation of m‐cresol to Toluene

**DOI:** 10.1002/cssc.202400685

**Published:** 2024-09-17

**Authors:** Roger Deplazes, Camila Abreu Teles, Carmen Ciotonea, Pardis Simon, Elias El Rassi, Jérémy Dhainaut, Maya Marinova, Nadia Canilho, Frédéric Richard, Sébastien Royer

**Affiliations:** ^1^ CNRS Centrale Lille UMR 8181 – UCCS – Unité de Catalyse et Chimie du Solide Univ. Artois Université de Lille 59000 Lille France; ^2^ UMR 7285 Université de Poitiers Institut de Chimie des Milieux et Matériaux de Poitiers (IC2MP), rue Michel Brunet, BP633 86022 Poitiers France; ^3^ Unité de Chimie Environnementale et Interactions sur le Vivant (UCEIV) UR 4492 Université du Littoral Côte d'Opale 59140 Dunkerque France; ^4^ Université de Lille CNRS INRA Centrale Lille Université Artois FR 2638 - IMEC – Institut Michel-Eugène Chevreul 59000 Lille France; ^5^ Laboratoire Lorrain de Chimie Moléculaire (L2CM) UMR 7053 Université de Lorraine 54506 Vandœuvre-lès-Nancy France

**Keywords:** Lignin, HDO, m-cresol, Ni−Cu nanoparticles, SBA-15

## Abstract

Amidst concerns over fossil fuel dependency and environmental sustainability, the utilization of biomass‐derived aromatic compounds emerges as a viable solution across diverse industries. In this scheme, the conversion of biomass involves pyrolysis, followed by a hydrodeoxygenation (HDO) step to reduce the oxygen content of pyrolysis oils and stabilize the end products including aromatics. In this study, we explored the properties of size controlled NiCu bimetallic catalysts supported on ordered mesoporous silica (SBA‐15) for the catalytic gas‐phase HDO of m‐cresol, a lignin model compound. We compared their performances with monometallic Ni and Cu catalysts. The prepared catalysts contained varying Ni to Cu ratios and featured an average particle size of approximately 2 nm. The catalytic tests revealed that the introduction of Cu alongside Ni enhanced the selectivity for the direct deoxygenation (DDO) pathway, yielding toluene as the primary product. Optimal performance was observed with a catalyst composition comprising 5 wt.% Ni and 5 wr.% Cu, achieving 85 % selectivity to toluene. Further increasing the Cu content improved turnover frequency (TOF) values, but reduced DDO selectivity. These findings underscore the importance of catalyst design in facilitating biomass‐derived compound transformations and offer insights into optimizing catalyst composition for more selective HDO reactions.

## Graphical abstract

Bimetallic NiCu nanoparticles supported on SBA‐15 were prepared by the glycine assisted in situ autocombustion method and further evaluated for the hydrodeoxygenation (HDO) of m‐cresol toward toluene in a continuous low‐pressure gas‐phase process.

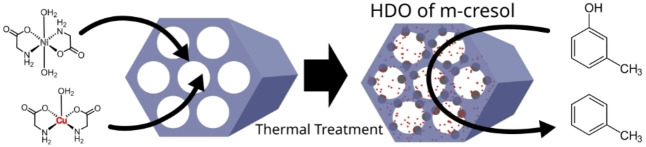

## Introduction

The scientific consensus on climate change is clear: Humanity needs to reduce its CO_2_ output from fossil hydrocarbons to net zero before the year 2050.^[1]^ In many sectors, fossil hydrocarbons can be substituted by other technology, or replaced by renewable alternatives. Batteries can then substitute gasoline and diesel, while fossil coal in the steel industry could be replaced by green hydrogen. However, in some sectors, fossil hydrocarbons are more difficult to replace. For instance, the high energy density of liquid hydrocarbons makes them difficult to replace in aviation and shipping fuels, as today′s batteries are too heavy for long distance flights and cannot store enough energy for intercontinental shipping. Where the hydrocarbons are not only used as fuel but also as raw material, this substitution becomes even more difficult, as is the case for the chemical and pharmaceutical industries. Given the size and importance of these three areas, it is an absolute necessity to find renewable alternatives to major fossil‐based chemical building blocks.

Biomass is the first alternative as it can serve as raw material for fuels and many chemical products. Fats, oils, sugars, and cellulosic biomass are already in wide use, whether chemically transformed, such as in bio‐diesel, or less processed, such as paper. Lignin, on the other hand, due to its complex composition, is mostly “thermally recycled”, *i. e*. it is incinerated for energy use.^[2]^ Lignin is also the second most abundant biopolymer on earth and a high‐quantity side product of the pulp and paper industry.^[2]^ It consists of interconnected aromatic monomers^[3]^ and could become a valuable source of aromatic hydrocarbons. Unfortunately for us, lignin is chemically highly complex and not straightforward to depolymerize.^[4]^

One way to achieve lignin depolymerization is through pyrolysis, during which the lignin is heated rapidly to high temperatures under the exclusion of oxygen, leading to partial depolymerization of the lignin and gasification of the products. The condensed pyrolysis gases are called ‘pyrolysis oils’ (or ‘bio‐oil’) and contain high quantities of water and oxygenated aromatics. The oxygenated compounds are responsible for the high reactivity of these pyrolysis oils, leading to corrosion and re‐polymerization.^[5]^ Therefore, a second processing step is needed reduce its oxygen content and thus stabilize the final oil, making it storable and enhancing its energy density. This process is called deoxygenation, or, if done in a hydrogen atmosphere, hydrodeoxygenation (HDO). Under given circumstances, the lignin‐derived monomers undergo mainly three different competing reactions: deoxygenation, hydrogenation, and C−C hydrogenolysis.^[6]^

The scarcity of green hydrogen necessitates an efficient process with as limited hydrogen‐consuming side reactions as possible. Therefore, a catalyst with a high selectivity for deoxygenation and low hydrogenation activity is needed. Different catalysts have demonstrated their activity for the HDO of lignin‐derived monomers and lignin pyrolysis oils. Among the most effective HDO catalysts reported are noble metals Rh,[^7^, ^8^] Ru,[^8^, ^9^, ^10^, ^11^] Pd,[^8^, ^9^, ^12^, ^13^, ^14^, ^15^, ^16^] and Pt.[^7^, ^8^, ^9^, ^14^, ^16^, ^17^, ^18^] Transition metals have been investigated, as well, with Ni showing the most promising results. Jin *et al*. tested different metallic Ni catalysts at 30 bar H_2_‐pressure and 210 °C (in liquid phase batch reactor with n‐decane as solvent) on their HDO activity with anisole and found, at full conversion, high amounts of deoxygenated but no aromatic products. Only reducing the H_2_‐pressure resulted in an increased selectivity for deoxygenated aromatic products, up to 53 % benzene selectivity over Ni/SiO_2_.^[19]^

Chen *et al*. have further shown that Ni can even compete with Pd and Pt catalysts in terms of toluene selectivity. They investigated the HDO performance of Ni/SiO_2_. At high conversion rates, a Ni catalyst showed a comparable deoxygenation vs. hydrogenation selectivity as the Pd catalyst and proved to be more selective than the Pt catalyst. Nevertheless, the activity of the Ni catalyst was several times lower compared to Pd and Pt, in part also due to the large particle size of Ni (25 nm vs. 10 nm for Pd).^[16]^

The influence of Ni particle size on the catalyst selectivity has been investigated by Teles *et al*. They tested 1, 3, and 9 nm Ni particles on SBA‐15 on the HDO of m‐cresol (continuous, atmospheric pressure, 300 °C), showing an increased selectivity for aromatic products when the Ni particles became smaller. It was proposed that active sites could increasingly be positioned on corners and edges favoring the DDO selectivity.^[20]^

The addition of a second transition metal and the subsequent formation of bimetallic particles can strongly influence the selectivity of a catalyst. Different authors have reported an improved deoxygenation selectivity of Ni catalysts when adding other transition metals. Adding large amounts of Co to Ni improved the catalyst activity and DDO selectivity in a study by Raikwar *et al*.^[21]^ Several authors have reported promising results for large bimetallic NiFe nanoparticles,[^22^, ^23^, ^24^] however, the present authors have not been able to confirm the improved activity and selectivity with small, finely dispersed nanoparticles.^[25]^ The most promising ones have been found with Cu: while monometallic Cu catalysts have not demonstrated high HDO activity, several authors reported the absence of any ring‐hydrogenation products.[^26^, ^27^, ^28^] The low activity of Cu catalysts stems from the Cu′s limited tendency to chemisorb H_2_. Pairing Cu, which exhibits low H_2_ affinity, with Ni, known for its high H_2_ affinity, seems, therefore, only consequential. NiCu bimetallic catalysts have already been applied successfully for the HDO of biomass pyrolysis oils from different sources: Guo et al. used NiCu/ZrO_2_ to upgrade algae pyrolysis oil, containing only low amounts of aromatic compounds, in a continuous gas phase reactor (350 °C, 20 bar H_2_).^[29]^ Boscagli et al. used NiCu/Al_2_O_3_ to treat wheat straw pyrolysis oil in a batch reactor (250 °C, 130–180 bar H_2_), however, the complex reaction mixture does not allow for a conclusion as to the different reactions that occurred.^[30]^

In the literature, NiCu bimetallic catalysts have also been used for the HDO of different models for lignin‐derived monomers. One study successfully used NiCu catalysts on SiO_2_, CeO_2_, and mixed supports for the liquid phase HDO of vanillin (batch, 150 °C, 25 bar at RT).^[31]^ Another study used NiCu catalysts on MCM‐41 and Ti‐MCM‐41 for the HDO of guaiacol (batch, 260 °C, 40–100 bar H_2_), achieving a higher activity on the Ti‐MCM‐41 support, but showing a higher selectivity towards aromatics for NiCu/MCM‐41.^[32]^ Vandevyvere et al. prepared Ni−Cu catalysts supported on amorphous SiO_2_ and γ‐Al_2_O_3_, containing mostly large particles (Cu>60 nm, NiCu>8 nm, NiO~4 nm) and applied them to the gas phase HDO of anisole at 200 °C and 5 bar pressure.^[33]^ The results show the almost complete hydrogenation of the aromatic ring and relatively low deoxygenation selectivity. Other examples for the application of NiCu bimetallic catalysts in HDO of anisole exist,^[34]^ but seem usually to concern large nanoparticles or even bulk alloys. Recently, copper catalysts and derived bimetallic formulations catalysts have also been proposed for other applications related to the valorization of biomass such as oxidation and reduction of platform molecules,[^35^, ^36^, ^37^] aldol condensation of furfural,^[38]^ besides the catalytic depolymerization of lignin.^[39]^

As of now, there are no significant results reported on continuous gas phase HDO of lignin‐derived monomers using finely dispersed NiCu nanoparticles. In the present study, a series of SBA‐15‐supported bimetallic NiCu catalysts with different Ni/Cu ratios were prepared by glycine‐assisted combustion‐impregnation, producing mostly nanoparticles of around 2 nm. The resulting bimetallic catalysts were tested on their catalytic performance in the gas phase HDO of m‐cresol using a fixed bed reactor at 300 °C and under atmospheric pressure. These results were lined up and compared to a monometallic Ni/SBA‐15 catalyst.

## Results and Discussion

### Catalyst Characterization

**Figure 1:**
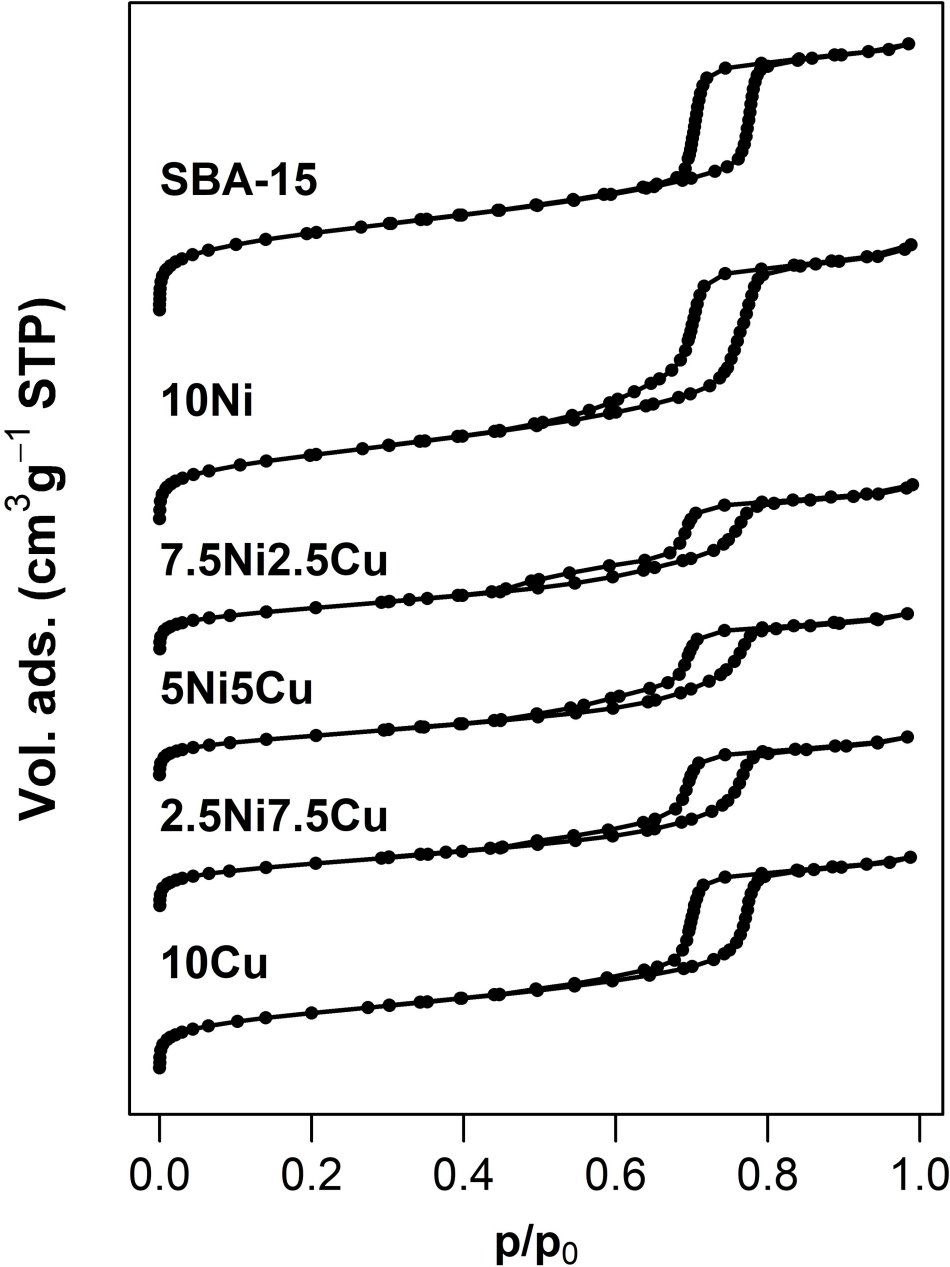
N_2_‐physisorption isotherms of the support (SBA‐15) and the calcined NiCu/SBA‐15 catalysts with a combined metal amount of 10 wt.%.

The N_2_‐physisorption isotherms of the catalysts and the bare silica support are given in Figure 1. The isotherms of all materials show the typical type IV shape of ordered mesoporous materials. The H1 hysteresis between p/p_0_=0.6 and 0.8 is typically obtained for mesoporous materials with a narrow pore size distribution.

The hysteresis window of the pure SBA‐15 shows parallel and desorption branches, indicating a highly ordered arrangement of cylindrical channels, giving rise to a honeycomb‐like 2D structure. The monometallic materials, 10Ni and 10Cu maintained the basic rhombic shape of the hysteresis. However, the desorption isotherm is not congruent with the adsorption isotherm at lower relative pressures (p/p_0_<0.7). This is most likely due to the presence of metal oxide particles inside the tubular pores leading to constrictions and pore plugging.^[40]^ The presence of such confined particles causes a partial blockage of the mesopore access thus inducing a decrease in the surface area and pore volume. The plugging effect is more evident for the bimetallic samples (7.5Ni2.5Cu, 5Ni5Cu, and 2.5Ni7.5Cu) which lose their rhombic shape. Furthermore, the congruency of the adsorption and the desorption isotherms becomes even worse below a relative pressure of p/p_0_=0.7. In the case of 7.5Ni2.5Cu and 5Ni5Cu, the isotherms almost open a second hysteresis window, suggesting the appearance of a second, smaller, pore fraction. Textural information extracted from the isotherms is summarized in Table 1. The bare support SBA‐15 showed a surface area of 799 m^2^ g^−1^. After the introduction of the metal oxide particles, both the BET surface area (S_BET_) and the microporous area (S_μ_), decreased, however to a different extent. For the 10Ni sample, the decrease in pore volume and S_BET_ was less prominent compared to the S_μ_, indicating that NiO nanoparticles are mainly confined within or at the mouth of the micropores of the walls. This is expected for materials prepared by the melt infiltration method during which the SBA‐15 still contains the porogen.^[41]^ In the case of 10Cu and bimetallic NiCu samples, S_BET_ showed a reduction of 41–44 %, and the decrease in pore volume corroborates the fact that there are particles inside the mesopores causing a partial plugging and creating a secondary porosity, as also evidenced for the slight decrease in the pore diameter. BJH analysis of the desorption branch suggests for 7.5Ni2.5Cu the existence of a small fraction of smaller pores with a diameter in the range of 3–4 nm (Figure S1, ESI).

**Table 1:** Physicochemical properties of the SBA‐15 support and the NiCu catalysts: metal content (ICP‐OES), porosity (N_2_‐physisorption), reducibility (H_2_‐TPR), and particle size (STEM).

	Metals			N_2_ physisorption				H_2_‐TPR	CO uptake (μmol g^−1^)	CO/Ni ratio^[h]^	d_M_^[i]^ (nm)
Sample	Ni^[a]^ (wt‐%)	Cu^[a]^ (wt‐%)	S_BET_[^b]^ (m^2^ g^−1^)	S_μ_^[c]^ (m^2^ g^−1^)	Vp^[d]^ (cm^3^ g^−1^)	Dp^[e]^ (nm)	Peak max.^[f]^ (°C)	Degree of red.^[g]^ (%)			
SBA‐15	–	–	799	296	1.07	7.3	–	–	–	–	–
10Ni	9.8	–	721	115	1.16	6.9	552	100	303	0.22	2.2
7.5Ni2.5Cu	7.1	2.3	470	54	0.69	6.2	298, 518	96	280	0.22	1.9
5Ni5Cu	4.9	4.4	447	55	0.69	6.4	235, 483	99	121	0.14	1.8
2.5Ni7.5Cu	2.2	7.3	472	78	0.72	6.6	227	71	80	0.19	1.9
10Cu	–	9.6	598	152	0.89	7.0	235	92	93	–	2.2

[a] Metal content determined by ICP‐OES analysis; [b] S_BET_ specific surface area calculated with BET equation; [c] S_μ_ microporous surface area determined with t‐plot method; [d] V_p_ pore volume at p/p_0_=0.95; [e] D_p_ average pore diameter calculated with BJH method applied to the desorption isotherm; [f] Maximum peak temperature recovered from TPR profiles; [g] Degree of reduction calculated from TPR results and based on the metal content from ICP‐OES; [h] CO/Ni molar ratio measured by CO chemisorptions [i] d_M_ average particle size calculated from HAADF images of the reduced catalysts using ImageJ software.

**Figure 2:**
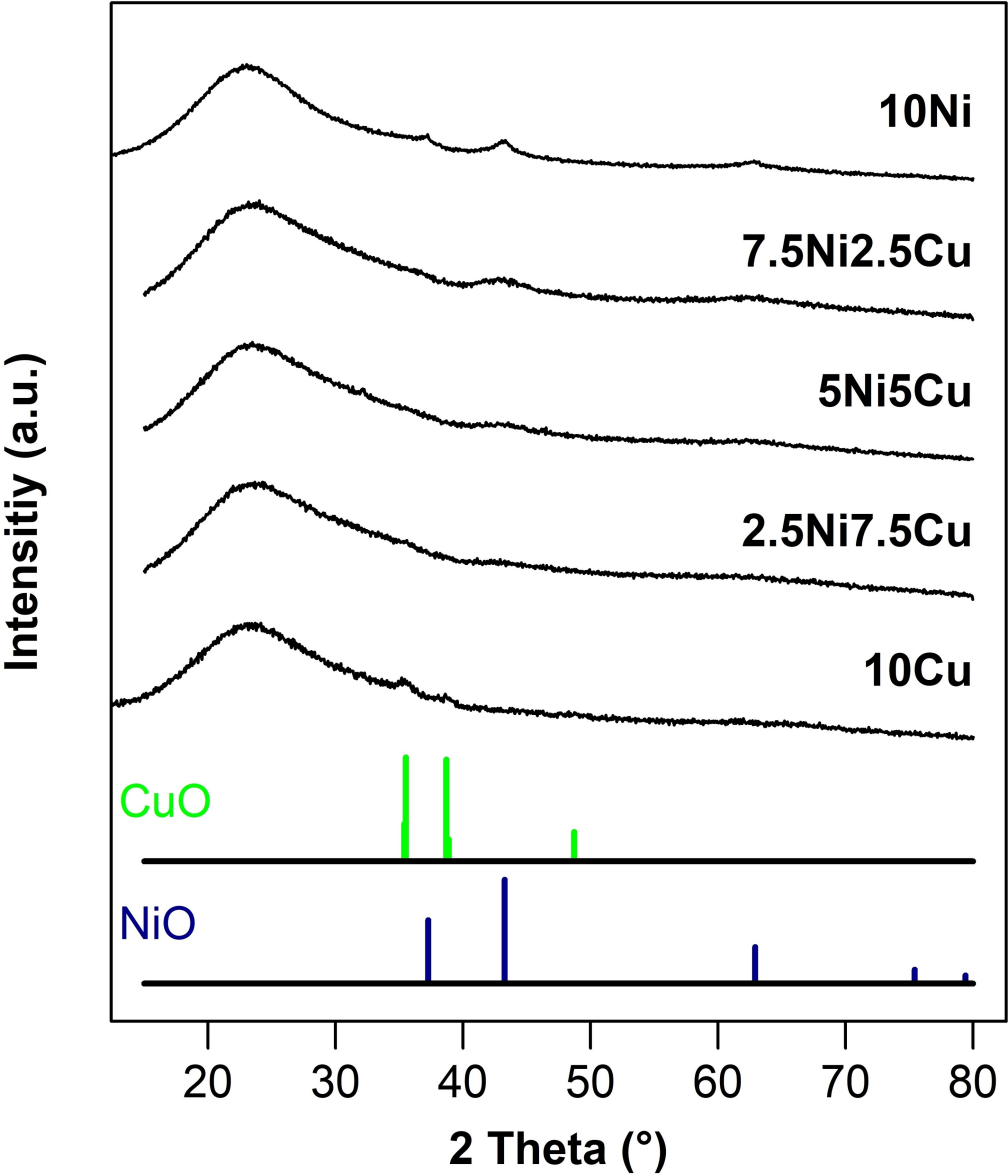
X‐ray diffraction patterns of the calcined NiCu/SBA‐15 catalysts with a combined metal content of 10 wt‐%.

Wide‐angle XRD patterns for the calcined samples and the respective NiO and CuO references are given in Figure 2. The only clearly distinguishable diffraction peaks can be found for the two monometallic samples. The diffractogram of the 10Ni shows three peaks at 2θ=37.3°, 43.3° and 62.9°, corresponding to the NiO crystalline phase (PDF file n° 47–1049). The diffractogram of 10Cu shows two main peaks at 2θ=35.5° and 38.8°, characteristic of the CuO crystalline phase (PDF file n° 45–0937). In both cases the peaks are broad and of very low intensity, indicating a low average crystallite size, *i. e*. below 3 nm as confirmed by STEM. The diffractograms of the bimetallic samples show no distinct peaks, but merely some flat “hills” in the same region as the NiO diffraction peaks, which is most pronounced for 7.5Ni2.5Cu, thus suggesting the presence of pure NiO nanoparticles. Given the absence of diffraction peaks corresponding to CuO in the mixed materials, it can be assumed that the CuO particles in these materials are either even smaller in size or amorphous.

**Figure 3:**
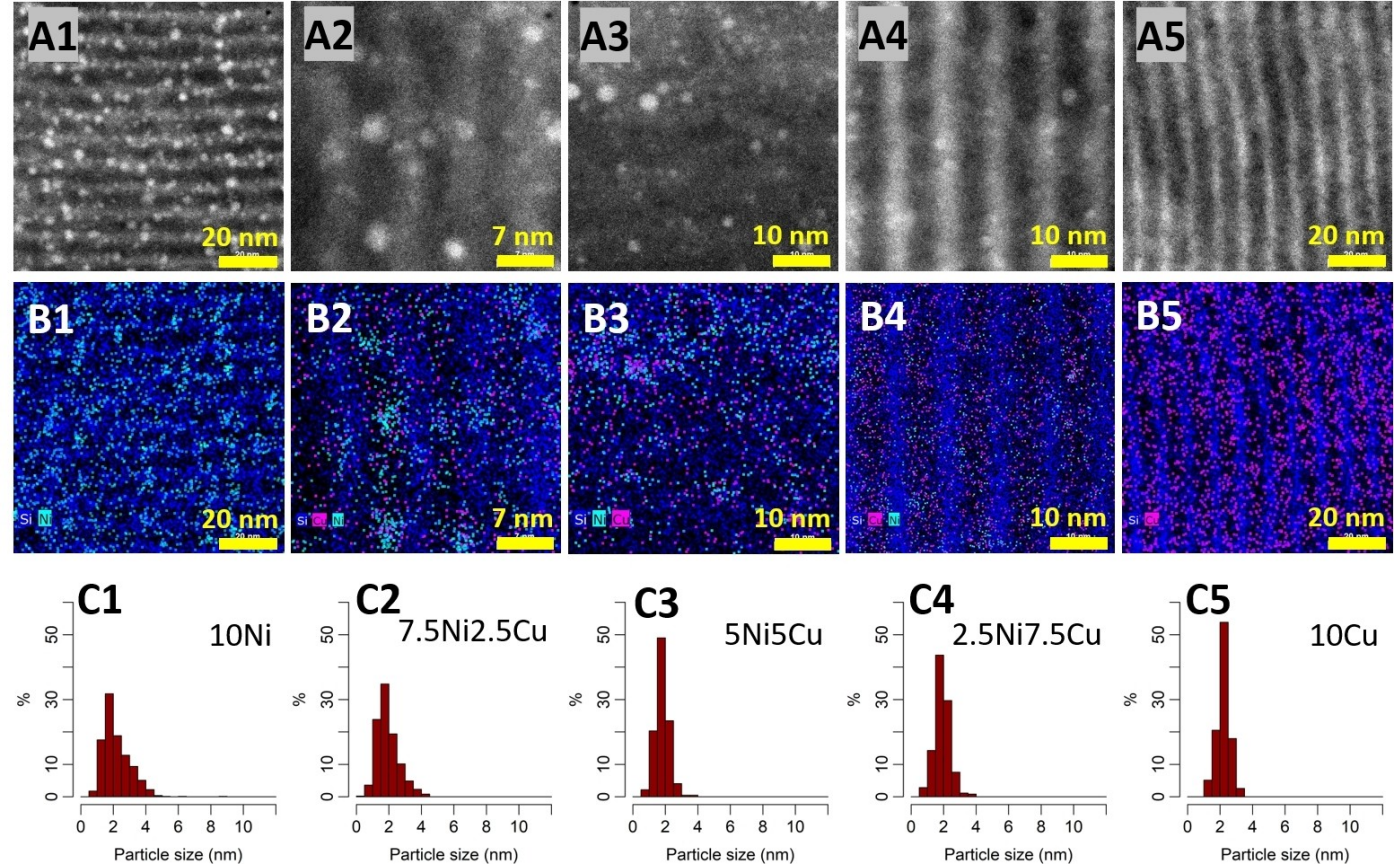
HAADF images (A), EDX mappings (B), and histograms of the particle size distribution (C) of the SBA‐15 supported NiCu catalysts. 1) 10Ni, 2) 7.5Ni2.5Cu, 3) 5Ni5Cu, 4) 2.5Ni7.5Cu, 5) 10Cu. All Ni‐containing catalysts were reduced in pure H_2_ at 600 °C for 1 h with a heat ramp of 10 °C/min.

This is largely confirmed by the STEM images shown in Figure 3. Before imaging, the samples were reduced for 1 hour under pure H_2_ at 600 °C. The HAADF images (Figure 3, A1–A5) clearly show the high dispersion of the metal nanoparticles on the SBA‐15 support, being partially located in the mesopores but mostly within their walls or at the mouth of micropores between the mesopores. The nanoparticle size distribution (Figure 3, C1–C5) showed an average size between 1.8 and 2.2 nm. This particle size is in accordance with the absence and/or very low intensity of diffraction peaks in the XRD and it suggests a resistance to significant sintering during the reduction at high temperature (600 °C). These results show the effectiveness of the synthesis methods used in this work to obtain very small nanoparticles with a high degree of dispersion after reduction. The STEM EDX maps (Figure 3, B1–B5) show the dispersion of the Ni and Cu throughout the SBA‐15 support and how they differ. It seems that Ni has a higher tendency to agglomerate than Cu, as is visible in the STEM EDX mapping shown in Figure B2–B4. Indeed, it is possible to identify areas with higher amounts of Ni, while no such area for Cu could be found. Comparing the images B2 (7.5Ni2.5Cu) and B4 (2.5Ni7.5Cu), one can easily see the difference, as B2 (high Ni, low Cu content) shows several distinguishable Ni nanoparticles, while B4 (low Ni, high Cu content) shows a very homogeneous Cu distribution, finely dispersed throughout the support porosity. However, based on the STEM EDX mappings it is not clear whether the nanoparticles in the bimetallic samples are alloyed or in the form of individual monometallic particles. In either case, the STEM EDX maps suggest a close contact between the two metals, even if this may only come from the high dispersion of Cu, potentially also covering the Ni nanoparticles. It needs to be added that in the two materials with pure Ni and pure Cu (10Ni and 10Cu), a small fraction of larger particles was observed, which are not representative of the average particle size estimation and are shown in the supplementary information (S2, ESI).

**Figure 4:**
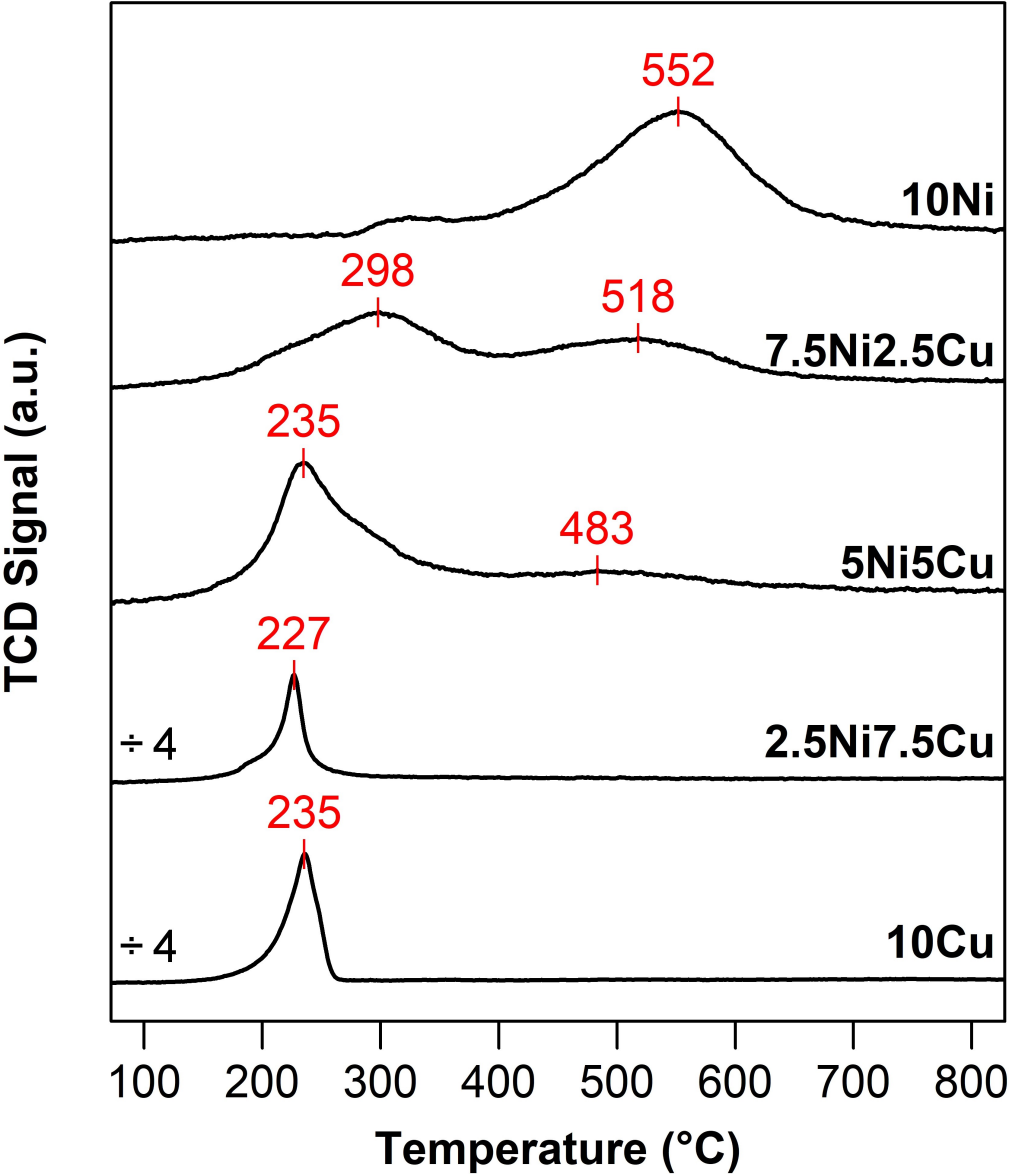
H_2_‐TPR profiles of the NiCu catalysts supported on SBA‐15. The temperatures at the peak maximum are labeled in red. Note that the intensity of 10Cu and 2.5Ni7.5Cu samples was divided by a factor of 4, in order to facilitate comparability

The reducibility of the NiCu‐based materials was analyzed by Temperature Programmed Reduction under H_2_ (H_2_‐TPR), of which the profiles are shown in Figure 4. For each metal, one peak is expected, denoting the reduction of Ni(II) to Ni(0) and Cu(II) to Cu(0). The 10Ni sample showed one main peak at 552 °C and one barely visible at around 300 °C. These peaks can be attributed to the reduction of the small, confined NiO particles, and the reduction of a few larger particles located on the external surface of the support, respectively.^[41]^ For the monometallic 10Cu sample, a narrow peak is observed at a lower temperature, *i. e*. 235 °C, somewhat lower than in the literature.[^27^, ^28^] The bimetallic samples showed completely different reduction profiles. Indeed, the introduction of Cu gradually shifted the reduction temperature of NiO to lower values, implying that the presence of Cu facilitates the reduction of NiO. This phenomenon is due to the hydrogen spillover from Cu to NiO, in which H_2_ is dissociated on the already reduced Cu, and active H species are spilled over to adjacent NiO, thus facilitating its reduction.^[42]^ For the 7.5Ni2.5Cu sample, two intense reduction peaks are observed at 298 and 518 °C, related to the reduction of Ni(II), either in direct contact with Cu or not, and a broad shoulder at around 230 °C due to the reduction of the small content of CuO. A further increase in the Cu content (by replacing Ni) leads to a shift towards lower temperatures of the first peak, while the second peak becomes smaller; for the 2.5Ni7.5Cu sample, the peak at the higher temperature is not even visible.

The reduction degrees are given in Table 1. The values, between 67 and 89 %, indicate that there is still a portion of unreduced Ni(II) and/or Cu(II) in the materials, even though the H_2_‐TPR was conducted up to high temperatures. This may be due to the presence of particles hardly accessible to H_2_, as they are inside the intra‐wall microporosity of the SBA‐15 support, and/or as multiple chained particles in the same pore. Taking these H_2_‐TPR results into consideration, prior to the catalytic test, all the Ni‐containing catalysts were reduced at 600 °C for 1 h and the monometallic Cu was reduced at 300 °C.

The density of accessible metallic sites on the catalyst surface was estimated by CO chemisorption, assuming a stoichiometric CO/Ni ratio of 1 (Table 1). The CO uptake gradually decreased by introducing Cu, from 303 μmol g^−1^ on the 10Ni catalyst to 93 μmol g^−1^ for the 10Cu sample. The lower CO chemisorption on Cu‐based catalyst can be explained by the filled d orbital of Cu, making it difficult to establish a σ bond with CO. As a consequence, we may assume that in the bimetallic samples, most of the CO is chemisorbed on the Ni surface and thus the values reflect the number of Ni sites at the catalyst surface. That said, a CO/Ni molar ratio was calculated and the values show that Ni dispersion, and consequently, Ni particle size, is almost constant (except for an abnormally slightly lower value for the 5Ni5Cu sample), which is in perfect agreement with the average particle size of the reduced samples measured in the TEM images.

The surface composition of the NiCu‐based catalysts was examined by XPS (Figures S3, 5, and 6). Table 2 summarizes the different Ni and Cu species fractions. Quantitative spectral fitting of Ni 2p_3/2_ spectra was made with two sets of multiplets corresponding to the Ni^2+^ state. For all of the calcined samples, the main peaks of Binding Energy (BE) were 853.7±0.1 eV and 856.1±0.1 eV which can be attributed to the presence of Ni^2+^ as NiO and Ni(OH)_2_ type species, respectively. The BE of Ni 2p_3/2_ main peak was 1.2 eV higher than that of pure Ni(OH)_2_ (BE=854.9 eV).^[43]^ This shift might be due to a strong interaction with the support.^[44]^ After reduction at 600 °C, a major part of the Ni^2+^ species were reduced, as evidenced by the appearance of an intense asymmetrical peak with BE at 852.6±0.1 eV associated with two low‐intensity satellites (BE at 3.7 eV and 6.0 eV above the main transition).

**Figure 5:**
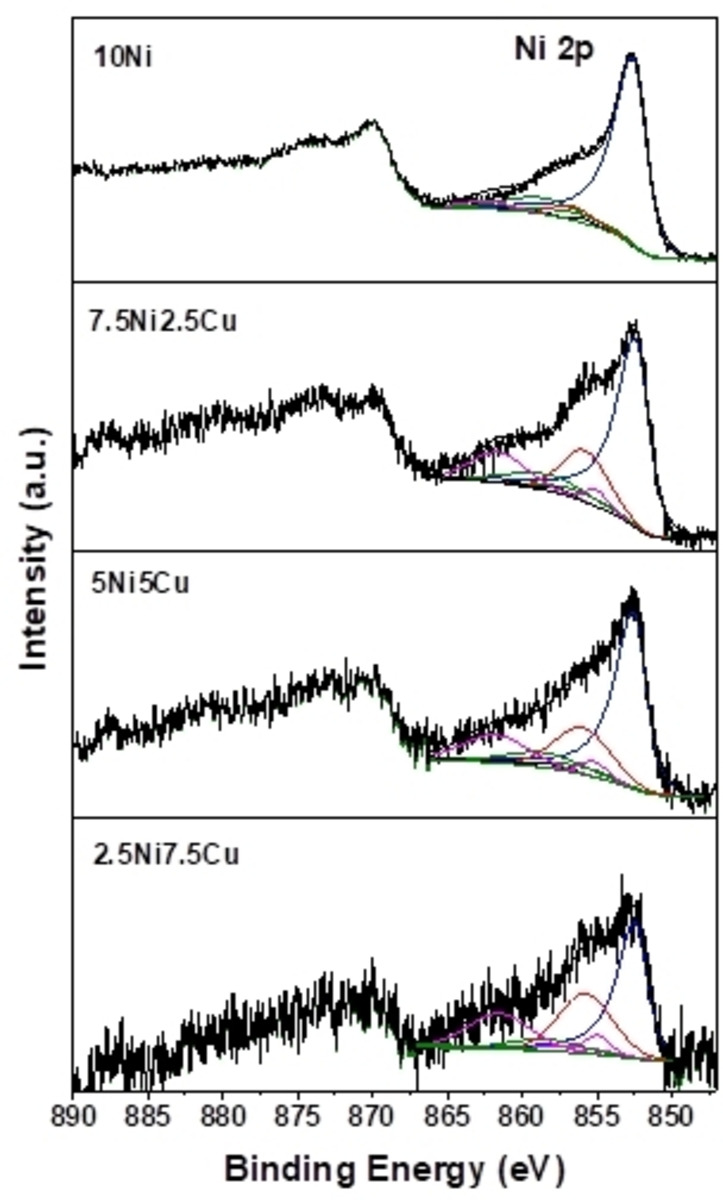
Ni 2p core level spectra as measured by XPS on reduced materials. Only the Ni 2p_2/3_ parts of the spectra were deconvoluted. Blue: Ni^0^; Green: satellite Ni^0^; Red: Ni^2+^; Pink: satellite Ni^2+^.

**Figure 6:**
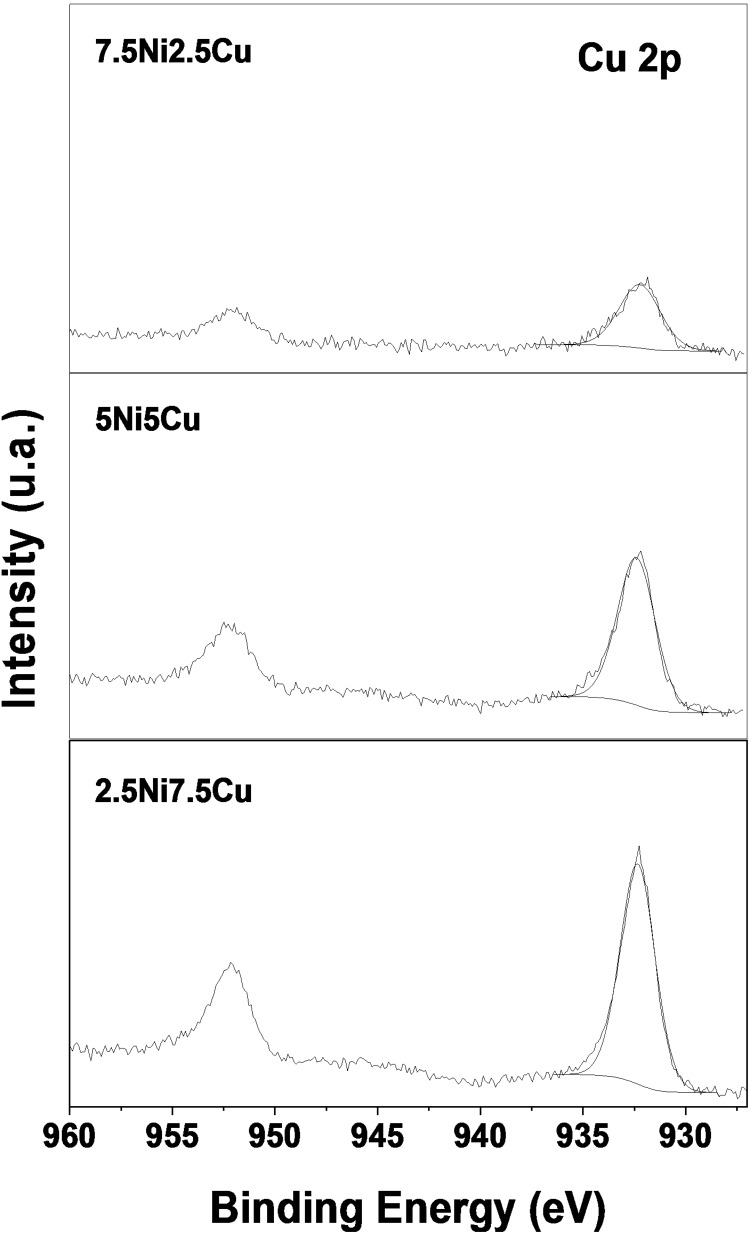
Cu 2p core level spectra as measured by XPS on reduced materials. Only Cu 2p_3/2_ parts of the spectra were decomposed.

**Table 2:** Relative surface composition of the NiCu catalysts calculated from XPS measurement. All values are given in at.%.

	Metals	Nickel			Copper		
Sample	Ni/Cu	Ni^0^	NiO	Ni(OH)_2_	Cu^0^	Cu(I)	Cu(II)
10Ni	–	–	9	91	–	–	–
10Ni_reduced	–	90	–	10	–	–	–
							
7.5Ni2.5Cu	2.3	–	11	89	–	91	9
7.5Ni2.5Cu_reduced	5.0	66	–	34	100	–	–
							
5Ni5Cu	0.9	–	10	90	–	89	11
5Ni5Cu_reduced	1.3	62	–	38	100	–	–
							
2.5Ni7.5Cu	0.4	–	–	100	–	81	19
2.5Ni7.5Cu_reduced	0.4	50	–	50	100	–	–

This contribution is characteristic of the presence of metallic Ni species. A remaining amount of Ni(OH)_2_ type was found, with different proportions depending on the content of Cu in the samples. This difference between the calcined and the reduced catalysts seems to decrease with increasing Cu‐content, which may influence the Ni reduction. Indeed, the fraction of reduced Ni decreases with the introduction of Cu (from 90 % in the 10Ni sample to only 50 % in the 2.5Ni7.5Cu sample, Table 2).

This last result agrees with the TPR result in which a reduction degree of 71 % was found for this catalyst with a higher content of Cu, in comparison to approximately 100 % for the others. In addition, the BE related to the remaining Ni(OH)_2_ and Ni^0^ species shifted towards low energy (BE decreased by 0.2 eV and 1.1 eV, respectively) for the Cu‐containing samples. This may be evidence of the formation of Ni−Cu solid solution or alloy. This shift has been observed by several authors in Ni_1‐x_Cu_x_ alloys when increasing the Cu content.[^34^, ^45^, ^46^]

Cu 2p_3/2_ spectra for all Cu‐containing samples exhibited a main peak localized at BE=933.2±0.1 eV which is characteristic of reduced copper species. Examination of the Cu LMM Auger peak allowed us to calculate the modified Auger Parameter (AP’) equal to 1847.1 eV. This low value suggests the presence of Cu(I) species, prior to reduction. An additional component is found at BE 935.5±0.2 eV, accompanied by a low‐intensity broad feature centered at BE around 944 eV attributed to a satellite peak. This second component is characteristic of the presence of a small amount of Cu(II) species. As the Cu 2p_3/2_ main peak for Cu(II) compounds is generally found at BE=933.8 eV for CuO and BE=934.7 eV for Cu(OH)_2_, the shift observed in BE may likely be due to strong interaction with the support as it was observed for the Ni species. Despite the thermal stability of Cu(II) species, the presence of Cu(II) as main species can be ruled out by the absence of a high intensity satellite structure which should be observed for Cu(II) compounds at approximately BE 7.4 eV higher than for the main Cu peak^[47]^ and that is not observed here. Therefore, the stabilization of Cu(I) instead of awaited Cu(II) could be associated to the decrease of Cu(II) species stability when associated to Ni(II). After reduction at 600 °C, the main peak was shifted to lower BE equal to 932.3±0.1 eV with increased AP’=1850.2±0.2 eV, which is consistent with the reduction of Cu cations to Cu^0^ species. In this case, due to the small chemical shift, it is, however, difficult to conclude on the presence of NiCu alloy species.^[48]^

### Catalytic Properties

The catalytic performances of monometallic 10Ni and 10Cu, as well as of the bimetallic NiCu catalysts, were investigated in the HDO reaction of m‐cresol at 300 °C under atmospheric pressure and 10 bar pressure.

**Scheme 1:**
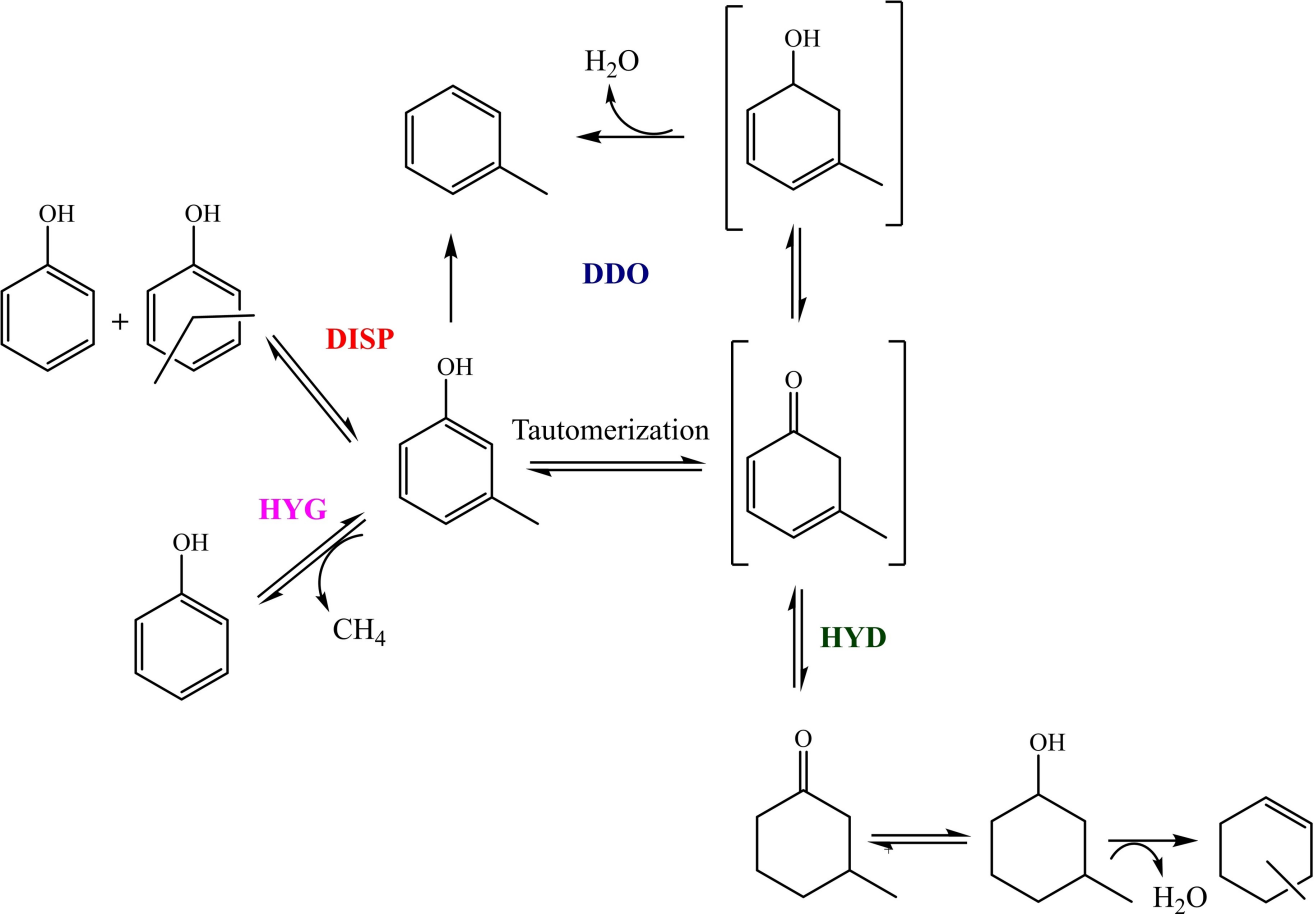
Reaction pathways proposed for the transformation of m‐cresol over the NiCu supported catalysts at 300 °C and atmospheric pressure.

Under atmospheric pressure, the product distribution obtained under comparable conditions of conversion (m‐cresol conversion close to 10 %) was measured by adjusting W/F (Table 3). For the 10Cu catalyst, the W/F required to obtain a measurable conversion (5 %) was significantly higher (17 times) compared to the respective monometallic 10Ni catalyst, indicating the poor activity of Cu under these experimental conditions (Table 4). Several works in the literature report that Cu/SiO_2_ catalysts were inactive in the HDO reaction. The authors attributed the low activity of Cu(0) to its lower hydrogenation ability, as well as, its low oxophilicity.[^8^, ^49^] However, in these published studies the Cu dispersion is poor, with particle sizes in the order of 16 nm. The preparation method used in the present work allowed us to synthesize materials with very small Cu particle sizes, on the order of 2 nm, and, hence, higher dispersion, explaining why conversion of m‐cresol can be observed over the 10Cu catalyst. Regarding product distribution, Cu alone favored the formation of 3‐methylcyclohexanone (m‐ONE; 51 mol %). Toluene (TOL) and 3‐methylcyclohexanol (m‐OL) were also observed in relatively high quantities (32 and 10 mol % in selectivity, respectively). On the other hand, for the 10Ni catalyst, TOL was the major product (71 mol %), showing the outstanding deoxygenation ability of Ni under the current reaction conditions. The m‐ONE and m‐OL were formed in minor amounts, as well as phenol and xylenols. With the addition of Cu, the selectivity into toluene slightly increased and the hydrogenated products (m‐ONE and m‐OL) remained almost constant. In addition, phenol and xylenol were not produced anymore. The formation of these products can be attributed to the engagement of four main parallel pathways, as highlighted in Scheme 1: (i) direct deoxygenation (DDO) to produce toluene, via direct cleavage of the C−O bond or by involving the formation of a tautomer intermediate (3‐methyl‐3,5‐cyclohexadienone), which can be hydrogenated, leading to an alcohol, a reactive intermediate, which in turn can be dehydrated into toluene; (ii) hydrogenation (HYD) of the tautomer aromatic ring producing the oxygenated products m‐ONE and m‐OL, which may be further transformed to methylcyclohexenes via dehydration reaction; (iii) hydrogenolysis (HYG) of the methyl group with formation of phenol and methane; (iv) disproportionation (DISP) in which phenol and xylenols are formed.

**Table 3:** Conversion of m‐cresol and the distribution of products obtained over the NiCu‐based catalysts at 300 °C and 1 atm.

Catalyst	W/F (g h mol^−1^)	Conversion (%)	Selectivity (%)					
			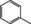	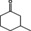	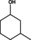	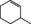	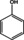	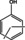
10Ni	6	10	73	14	2	1	6	2
7.5Ni2.5Cu	4	10	80	15	2	3	–	–
5Ni5Cu	10	17	85	11	2	2	–	–
2.5Ni7.5Cu	6	11	74	17	5	2	–	–
10Cu	105	5	32	51	10	7	–	–

**Table 4:** Reaction rates for the transformation of m‐cresol and for the main reaction pathways after 0.5 h on stream. TOT: total conversion; DDO: direct deoxygenation pathway; HYG: hydrogenation pathway.

Catalyst	r^[a]^ (mmol g_cat_^−1^ h^−1^)			r^[b]^ (mmol g_Ni_^−1^ h^−1^)			TOF^[c]^ (h^−1^)
	r_TOT_	r_DDO_	r_HYD_	r_TOT_	r_DDO_	r_HYD_	
10Ni	16.5	12.2	2.9	168	124	30	54
7.5Ni2.5Cu	26.4	21.0	5.3	372	295	71	94
5Ni5Cu	17.0	14.5	2.2	347	296	45	140
2.5Ni7.5Cu	18.5	13.8	4.7	841	630	214	231
10Cu	0.5	0.2	0.3	–	–	–	–

[a] constant rate calculated per gram of catalyst and [b] per gram of Ni. [c] Turnover frequency for m‐cresol conversion, considering only Ni as active sites.

This reaction scheme agrees with those already reported in the literature.[^8^, ^9^] The contribution of each of these reaction routes depends on the type of catalyst, particularly on the properties of the metallic phase and of the support used. For example, oxophilic metals, such as Ru and Fe, or oxophilic supports, such as ZrO_2_, TiO_2_, and Nb_2_O_5_, promote the DDO route instead of the HYD pathway, due to the strong interaction with the oxygen atom which weakens the C−O bond.[^9^, ^22^, ^50^, ^51^] On the other hand, less oxophilic metals such as Pt, Pd, or Ni mainly promote the HYD route, producing ketones and alcohols as main products.[^9^, ^22^] By introducing acid sites in the catalytic support, dehydration of the intermediate alcohols is favored, leading to the formation of alkenes, as well as, promoting the DISP pathway.[^15^, ^22^] Metals such as Ru, Ni, and Co are also able to promote hydrogenolysis (HYG), yielding undesirable methane formation.[^9^, ^52^] However, some strategies can be used to modify the behavior of such metals in the HDO reaction, by controlling metal particle size or alloying hydrogenating metals with oxophilic ones for example. Previously, it has been demonstrated how smaller Ni particles (<3 nm) promoted the DDO route, while larger particles favor both the HYD and HYG pathways.[^20^, ^52^] It was proposed that the metal oxophilicity depends on the extent of coordination of metal atoms. Thus, smaller particles, which contain more low‐coordination sites, such as edges and corners, are more active for the formation of toluene (DDO route), while larger particles presenting more high‐coordination sites, such as terrace, tend to promote ring hydrogenation (HYD route) and C−C bond hydrogenolysis (HYG). In the present work, we take advantage of two synthesis methods to control the metal particle size (all of the catalysts present small Cu and Ni nanoparticles of around 2 nm), to maximize catalyst activity and to improve the selectivity towards toluene.

**Figure 7:**
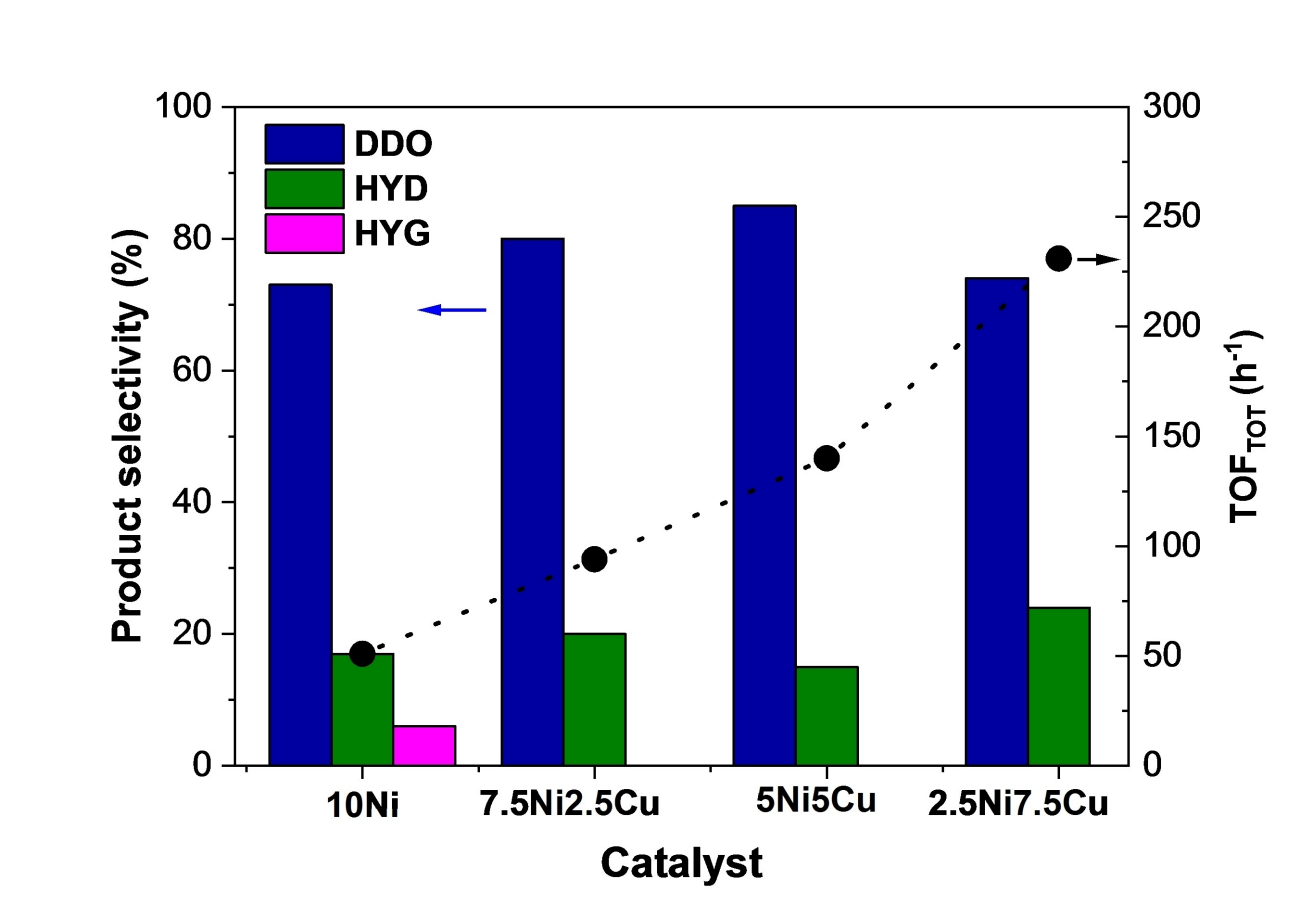
Products obtained in the main reaction pathways and TOF of m‐cresol conversion for the different catalysts.

The 10Ni catalyst favored the DDO pathway instead of the HYD route, associated to the presence of oxophilic small Ni nanoparticles active in C−O bond scission. With Cu adding, differences are observed regarding product distribution (Table 3, Figure 7). The presence of Cu(0) promoted the selectivity toward the DDO route (73 mol % for 10Ni, as compared to 85 mol % with 5Ni5Cu) and suppressed both HYG and DISP routes. There is hence an optimal amount of Cu (Ni/Cu=1) to maximize the formation of toluene. The selectivity into hydrogenated products (m‐ONE, m‐OL, and m‐ENEs) remained constant. A Ni−Cu phase with Cu enrichment breaks the continuity of the Ni surface, thus reducing the size of the Ni ensemble. As a consequence, the HYG products (phenol and methane) were suppressed. Note that for the 2.5Ni7.5Cu catalyst, the selectivity to hydrogenated products started to increase at the expense of toluene.

The experimental conditions, particularly H_2_ pressure, influence the catalytic activity and selectivity in reaction. Increasing the H_2_ pressure favors the formation of products obtained through the HYD route (Scheme 1). Then, under H_2_ pressure (30 bar), hydrogenated oxygenated compounds (alcohols and ketones) or alkanes were the main products from m‐cresol transformation whereas toluene was favored under atmospheric pressure, in the presence of Pd based catalysts.^[15]^ The catalytic properties of three catalysts (10Ni, 5Ni5Cu and 10Cu) were then evaluated under 10 bar at 300 °C using the same contact time (W/F ratio equal to 1.7 g h mol^−1^). As expected, H_2_ pressure favored the HYD route, methylcyclohexanol and methylcyclohexanone becoming the main products of the transformation, whereas the selectivity into toluene was low (6–11 % depending on the catalyst; vs. 32–85 % when reaction is at atmospheric pressure) (Table S1).

To understand how the presence of Cu affects the catalytic properties of Ni nanoparticles, the reaction rates (total and for each main HDO route) for the transformation of m‐cresol are reported in Table 4 for atmospheric pressure reaction and in Table S2 for reaction conducted under 10 bar. By replacing 25 % of Ni with Cu, the DDO rate almost doubled compared to monometallic Ni (from 12.2–21 mmol g_cat_^−1^ h^−1^) and increased by 105 times compared to monometallic Cu. However, by further increasing the content of Cu, the DDO rate decreased, despite being still slightly higher compared to the one obtained for monometallic Ni. As observed in Tables 4 and S2, the activity of the Cu(0) nanoparticles was negligible compared to Ni(0) nanoparticles. Hence, reaction rates and turnover frequency (TOF) were calculated considering only the Ni as an active metallic phase. As can be seen in Figure 7, despite no remarkable change in product selectivity, the intrinsic activity of Ni significantly increased with the introduction of Cu. Indeed, the DDO rate continuously increased, with the 2.5Ni7.5Cu catalyst presenting an outstanding activity at atmospheric pressure.

Very few works reported supported NiCu catalysts in the HDO reaction of phenolic compounds, still less in the gas phase and with well‐dispersed NiCu catalysts. For instance, the effect of the Ni/Cu metal ratio, supported on silica, was investigated for the HDO of anisole and m‐cresol.[^33^, ^49^] Despite performing the reactions at significantly different temperatures and H_2_ pressures, in both studies, the monometallic Cu‐catalyst was found to be inactive, with the activity being dependent on the Ni content, highlighting the positive effect of the formation of NiCu alloys. It was suggested that the addition of Cu facilitated the reduction of Ni species and enhanced metal dispersion, thus improving catalyst activity.^[33]^ Wang et al. Investigated a series of NiCu/SiO_2_ in the HDO reaction of m‐cresol at 350 °C under atmospheric pressure.^[49]^ DDO and HYG routes were favored, depending on the type of catalyst used. Monometallic Ni favored HYG, producing phenol and methane as major products, and the introduction of Cu promoted the formation of toluene, agreeing with our results. Regarding catalyst activity, increasing the Cu/Ni molar ratio from 0 to 1 reduced the TOF of HYG by 10 times while increasing the toluene formation by around 3 times. However, the TOF of m‐cresol conversion slightly decreased from 192 to 184 h^−1^, which lies between the TOF of 5Ni5Cu and 2.5Ni7.5Cu (Table 4). As Cu was inactive, these trends indicate that the presence of Cu vicinal to Ni has a minor effect on the overall HDO activity of Ni, however, it modified the preferred reaction pathway.

In our work, the small nanoparticles are in principle favorable for C−O bond cleavage, regardless of the Cu presence, which explains the constantly high selectivity to toluene. The partial replacement of Ni by Cu facilitated the reduction of Ni and diluted the surface Ni, thus increasing the number of exposed active sites and consequently the intrinsic catalyst activity.

## Conclusions

In this study, two preparation methods were used to produce SBA‐15‐supported Ni, Cu, and NiCu bimetallic catalysts with particle sizes of around 2 nm. Further, their performance for the gas‐phase HDO of m‐cresol to aromatic compounds was evaluated.

The 10Ni catalyst showed already a high activity and a reasonably good selectivity towards toluene, while the 10Cu catalyst displayed a significantly lower conversion rate. The mixed bimetallic catalysts 7.5Ni2.5Cu, 5Ni5Cu, and 2.5Ni7.5Cu demonstrated an improved activity when normalized for the Ni content, as well as, the suppression of the C−C hydrogenolysis reaction. Furthermore, they showed an equal or higher selectivity to toluene (DDO product) than 10Ni, reaching the best DDO selectivity with 85 % for the 5Ni5Cu catalyst. Further increasing the Cu content led to another increase of the TOF per active site, but also resulted in a lower DDO selectivity.

These results clearly show the great potential that lies in the use of NiCu bimetallic catalysts for the HDO of lignin‐derived monomers to aromatics. Moreover, the observed trade‐off between increasing Cu content, elevated TOF, and reduced DDO selectivity provides valuable insights into catalyst optimization strategies. As we navigate the transition towards sustainable energy sources, the findings presented here contribute to the development of catalysts free of noble metal for the conversion of biomass‐derived compounds.

Looking ahead, further investigations into the mechanistic aspects and scalability of these catalysts will be essential to bridge the gap between laboratory‐scale studies and practical application. The nuanced understanding gained from this research paves the way for continued advancements in green chemistry, offering a pathway towards a more sustainable and diversified energy landscape.

## Experimental

### Materials

All chemicals required for the preparation of the support and the catalysts were used as received: Si(OC_2_H_5_)_4_ (TEOS, 98 wt.%, Sigma‐Aldrich), non‐ionic triblock copolymer Pluronic P123 (poly(ethylene oxide)‐block‐poly(propylene oxide)‐block‐poly(ethylene oxide), PEO_20_PPO_70_PEO_20_, MW=5800, Aldrich), hydrochloric acid (HCl, 37 wt.%, CARLO ERBA Reagents), nickel nitrate (Ni(NO_3_)_2_ ⋅ 6H_2_O, 98 wt.%, Alfa Aesar), copper nitrate (Cu(NO_3_)_2_ ⋅ 3H_2_O, 99 wt.%, Acros Organics).

### Support Preparation

SBA‐15 was synthesized in a single large batch, in a modified version of the original by Zhao et al.^[53]^ Deionized water (6.5 L), HCl (37 %; 1 L), and Pluronic P123 (200 g) were firstly mixed in a 10 L glass reactor and stirred for 24 h at 40 °C. Then, the silicon source, TEOS (480 mL), was added dropwise and the solution stirred at 40 °C for another 24 hours. Then, the temperature was increased to 96 °C and kept for 48 hours. After cooling to room temperature, the precipitate was filtered, washed with deionized water, and dried at 80 °C overnight. A further calcination step (550 °C, 6 h, 1.5 °C min^−1^) was applied to the SBA‐15 when necessary.

### Catalyst Preparation

The catalysts were prepared by using two different methods. The 10Ni catalyst supported on SBA‐15 was prepared by Melt Infiltration (MI) method, as previously reported.^[41]^ The appropriate amount (to reach 10 wt.% metal in the final material) of the precursor Ni(NO_3_)_2_ ⋅ 6H_2_O was incorporated into the non‐calcined SBA‐15 by gentle grinding at ambient conditions. The resulting powder was then treated in a PTFE autoclave at 57 °C for 4 days. The material was finally calcined at 500 °C for 6 h (heating ramp of 1.5 °C min^−1^).

The Cu‐containing catalysts were prepared by the In Situ Auto Combustion (ISAC) method using glycine as a complexing agent.[^54^, ^55^] Appropriate amounts of the precursors (Cu(NO_3_)_2_ ⋅ 3H_2_O and Ni(NO_3_)_2_ ⋅ 6H_2_O) were dissolved in around 20 mL of H_2_O and glycine was added (4 eq. with respect to the metals). After complete dissolution, calcined SBA‐15 was added and the mixture was left for 2 hours. The slurry was then dried on a moderately hot heating plate before heating the powder rapidly above the auto‐combustion temperature of the glycine (233 °C), leading to partial combustion. A calcination step followed (500 °C, 6 h, 1.5 °C min^−1^). The materials were prepared with varying content of Ni and Cu (Ni/Cu being 0/10, 2.5/7.5, 5/5, 7.5/2.5) keeping the total content of Ni−Cu at 10 wt.%. The catalysts were named *x*Ni*y*Cu, with x and y giving the specific per wt.% of the subsequent metal.

### Catalyst Characterization

Characterization was done analogous to previous work:^[25]^ Ni and Cu contents were determined by inductively coupled plasma optical emission spectroscopy (ICP‐OES) on an Agilent′s 5110 Vertical Dual View ICP‐OES equipped with a OneNeb nebulizer instrument. Before analysis, the samples were digested in concentrated nitric acid and hydrofluoric acid on a microwave heating system.

Textural properties were determined by N_2_‐physisorption analysis at −196 °C on a Micromeritics Tristar III instrument. Before analysis, the samples were degassed under a dynamic vacuum at 150 °C for 3 h. The specific surface area was determined using the multipoint BET algorithm, the mesopore size distribution was determined by applying the BJH equation to the desorption branch, and the pore volume was determined at *p/p_0_* =0.95, on the adsorption branch. The micropore surface and microporous volume were determined with the t‐plot method.

Powder X‐ray diffraction (XRD) analysis was performed using a Bruker X‐ray AXS D8 Advance X‐ray diffractometer in Bragg‐Brentano geometry configuration. The XRD patterns were recorded with Cu K_α_ radiation (*λ*=0.154 nm) in the range *2θ*=15–80° with a step size of 0.05° and dwell time of 2 s. Phase identification was made by comparison with the ICDD database.

Support morphology and metal dispersion state were analyzed by scanning transmission electron microscopy (STEM) on a TITAN Themis 3007 S/TEM equipped with a high brightness Schottkey field emission gun, a probe aberration corrector, super‐X detector system with four windowless silicon drift detectors for electron dispersive X‐ray spectroscopy (EDX) and several annular dark field detectors. The experiments have been performed at 300 kV with a semi‐convergence angle of about 20 mrad, probe size of the order of 500 pm, and probe current at around 100 pA. For high‐angle annular dark field (HAADF) imaging, the camera length has been chosen such that the collection angles were between 50 and 200 mrad. The EDX mapping has been obtained in multi‐frame spectrum imaging mode with a dwell time per pixel of about 15 μs and continuously scanning frames up to a total acquisition time of about 15–20 minutes. Before analysis, the samples were reduced at 600 °C for 1 h (temperature ramp of 10 °C min^−1^) and then embedded in polymeric resin, sliced into 50 nm‐thick sections using an ultramicrotome, and deposited on a carbon grid for analysis.

The reducibility of the catalysts was investigated by temperature‐programmed reduction using H_2_ as a reducing agent (H_2_‐TPR) on a Micromeritics Autochem II 2920 device. Before analysis, the solids were heated to 500 °C under 50 mL min^−1^ air flow (10 °C min^−1^). After cooling down to room temperature, the H_2_‐TPR was performed up to 900 °C using a 50 mL min^−1^ flow of 5.0 vol.–% H_2_ in Ar, and a temperature ramp of 5 °C min^−1^. The consumption of H_2_ was measured by a TCD detector.

The metallic dispersion was estimated by CO chemisorption analysis. The samples were previously reduced at 600 °C (5 °C min^−1^), under pure hydrogen flow (30 mL min^−1^) during 1 h. Then, the reactor was cooled down to the chemisorption temperature (30 °C) under helium (30 mL min^−1^) and successive pulses of pure CO (0.465 mL) were injected each 3 min. CO uptake was quantified by using a gas phase chromatograph equipped with a TCD detector and a Porapak Q column.

The surface composition of the calcined and the reduced samples was evaluated by X‐ray photoelectron spectroscopy (XPS) analysis. The analyses were performed on a Kratos Analytical Axis Ultra DLD spectrometer, equipped with a monochromatic Al K_α_ X‐ray source (1486.6 eV) operating at 225 W (15 kV, 15 mA). The charge neutralizer system was used for all acquisitions, with a Pass Energy of 20 eV and a step size of 0.05 eV. Fresh samples were analyzed under ultra‐high vacuum (UHV) with an instrument base pressure of 5 ⋅ 10^−10^ torr. The activated samples were reduced under 50 mL min^−1^ of pure H_2_ at 600 °C (5 °C min^−1^, 1 h) in a high‐temperature cell attached to the XPS instrument. Afterwards, the samples were cooled under H_2_, and then transferred under UHV to the XPS analysis chamber, without re‐exposure to air. Binding energies (BE) were referenced to the unresolved Si 2p doublet for SBA‐15 positioned at 103.5 eV. Simulation of the experimental peaks was carried out with CasaXPS software using mixed Gaussian (70 %)/Lorentzian (30 %) peaks except for metallic Ni, where LA (1.1, 2.2, 10) line shape was used. Semiquantitative analysis was performed after the subtraction of Shirley‐type backgrounds.

### HDO of m‐cresol

The HDO reaction of m‐cresol was carried out in a vertical fixed‐bed flow reactor system, operating at 300 °C and atmospheric pressure. The H_2_/m‐cresol molar ratio was fixed at 90. Before the reaction, the catalysts were reduced *in situ* for 1 h under pure H_2_ (50 mL min^−1^) at 600 °C and a temperature ramp of 5 °C min^−1^. The reactant feedstock, composed by m‐cresol as reactant (7 mol‐%), decane as internal standard (3 mol‐%), and heptane as the solvent, was introduced at the top of the reactor using a syringe. W/F values, defined as the ratio of catalyst weight (in g) and m‐cresol molar flow rate (in mol h^−1^) were to obtain comparable levels of conversion (around 10 %). The reactor outlet was connected to a cold‐trap kept at 5 °C using a circulator Huber minichiller in order to condense any unconverted reactant and products. The first liquid sample was collected after 30 min of reaction and analyzed by a Varian 430 chromatograph equipped with a DB‐5 capillary column and a flame‐ionization detector (FID). The conversion of m‐cresol and the product selectivity were calculated using Equations (1) and 2:

(1) $$X\;\left(in\;\%\right)=\frac{{n^{0}}_{m-cresol}\;-\;n_{m-cresol}}{{n^{0}}_{m-cresol}\;}\times 100$$

(2) $$S_{i}\;\left(in\;mol\;\%\right)=\frac{n_{i}}{{n^{0}}_{m-cresol}\;-\;n_{m-cresol}\;}\times 100$$

where *n* ^*0*^_*m‐cresol*_ and *n*_*m‐cresol*_ are the number of moles of m‐cresol initially and after 30 min of reaction, respectively; *n_i_* is the number of moles of a given product *i*.

Assuming a pseudo‐first‐order reaction rate, the total kinetic rate constant (*k_TOT_* in mmol⋅g_Ni_^−1^ h^−1^) and the various kinetic rate constants for the different reaction pathways were calculated using Equations (3) and (4): 34>

(3) $$k_{TOT\;}\left(in\;mmol\cdot {g_{Ni}}^{-1}\cdot h^{-1}\right)=\frac{-F\cdot ln\;(1-X)}{w\cdot \left(\%Ni\right)\;}$$

(4) $$k_{y}\left(in\;mmol\cdot g^{-1}\cdot h^{-1}\right)=k_{TOT}.\;S_{y}$$

where *k_TOT_* is the total kinetic rate constant determined for the global conversion of m‐cresol and *k_y_* is the kinetic rate constant for each reaction pathway *y*, *X* is the m‐cresol conversion, *F* is the m‐cresol flow rate (in mmol h^−1^), *w* is catalyst weight (in g), %Ni is the Ni content as given by ICP‐OES and *S_y_* is the selectivity to the different products *y*: toluene for the direct deoxygenation route; 3‐methylcyclohexanone, 3‐methylcylohexanol, methylcyclohexene isomers for the hydrogenation route; and phenol for the hydrogenolysis route.

The turnover frequency (TOF, in h^−1^) values were calculated with Equation (5): 5

(5)

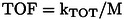




where *M* is the CO uptake (in mmol g^−1^) obtained from chemisorption measurements.

## Conflict of Interests

The authors declare no conflict of interest.

1

## Supporting information

As a service to our authors and readers, this journal provides supporting information supplied by the authors. Such materials are peer reviewed and may be re‐organized for online delivery, but are not copy‐edited or typeset. Technical support issues arising from supporting information (other than missing files) should be addressed to the authors.

Supporting Information

## Data Availability

The data that support the findings of this study are available from the corresponding author upon reasonable request.
