# Supplementary material for: SBA‐15 Supported Ni‐Cu Catalysts for Hydrodeoxygenation of m‐cresol to Toluene
Source: ChemSusChem. 2024 Sep 17;18(1):e202400685. doi: 10.1002/cssc.202400685 (PMC11696214; doi:10.1002/cssc.202400685)
Supplement: Supplementary file 1 — Supporting Information [file CSSC-18-e202400685-s001.pdf]

ChemSusChem

Supporting Information

SBA-15 Supported Ni-Cu Catalysts for Hydrodeoxygenation of m-cresol to Toluene

Roger Deplazes, Camila Abreu Teles, Carmen Ciotonea, Pardis Simon, Elias El Rassi, Jérémy Dhainaut, Maya Marinova, Nadia Canilho, Frédéric Richard,* and Sébastien Royer*

Supplementary Information

SBA-15 supported Ni-Cu catalysts for hydrodeoxygenation of m-cresol to toluene

Roger Deplazes, Camila Abreu Teles, Carmen Ciotonea, Pardis Simon, Elias El Rassi, Jérémy Dhainaut, Maya Marinova, Nadia Canilho, Frédéric Richard, Sébastien Royer

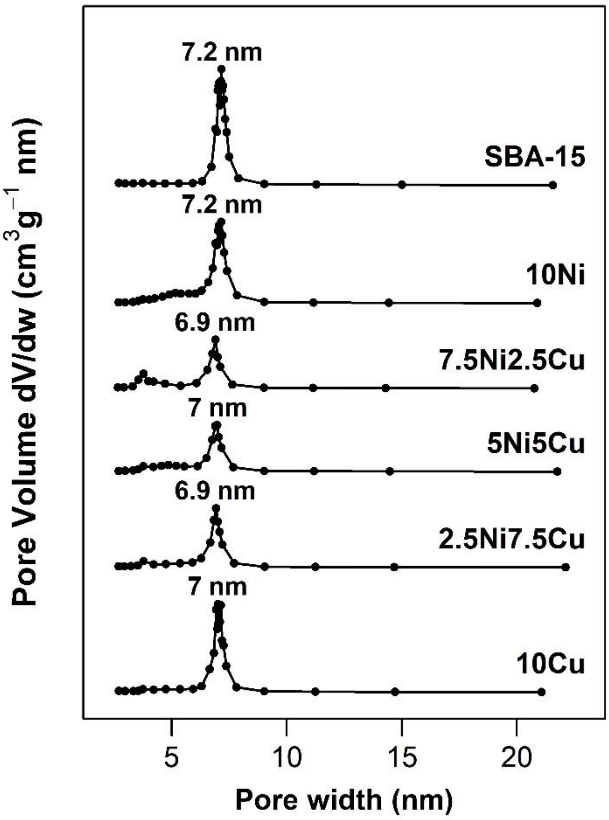

Figure S1. Pore width distribution as calculated by application of the BJH method on the desorption isotherm of the bare SBA-15 support and the NiCu/SBA-15 materials after calcination.

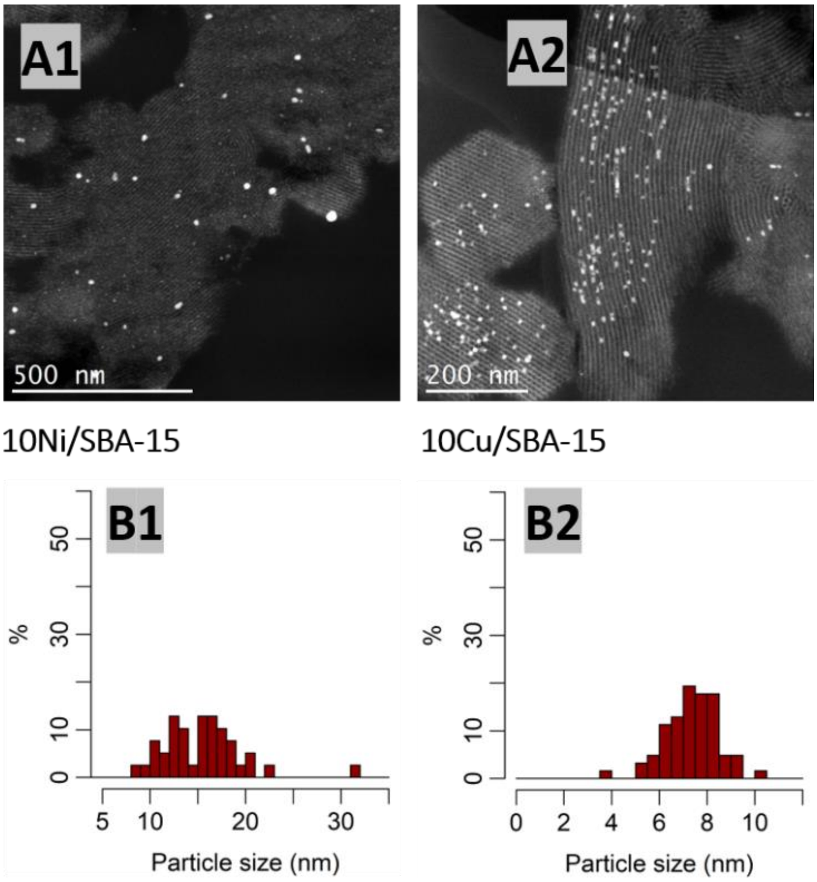

Figure S2. HAADF images of 10Ni/SBA-15 (A1) and 10Cu/SBA-15 (A2) showing the large particles with the corresponding particle size distributions (B1 and B2).

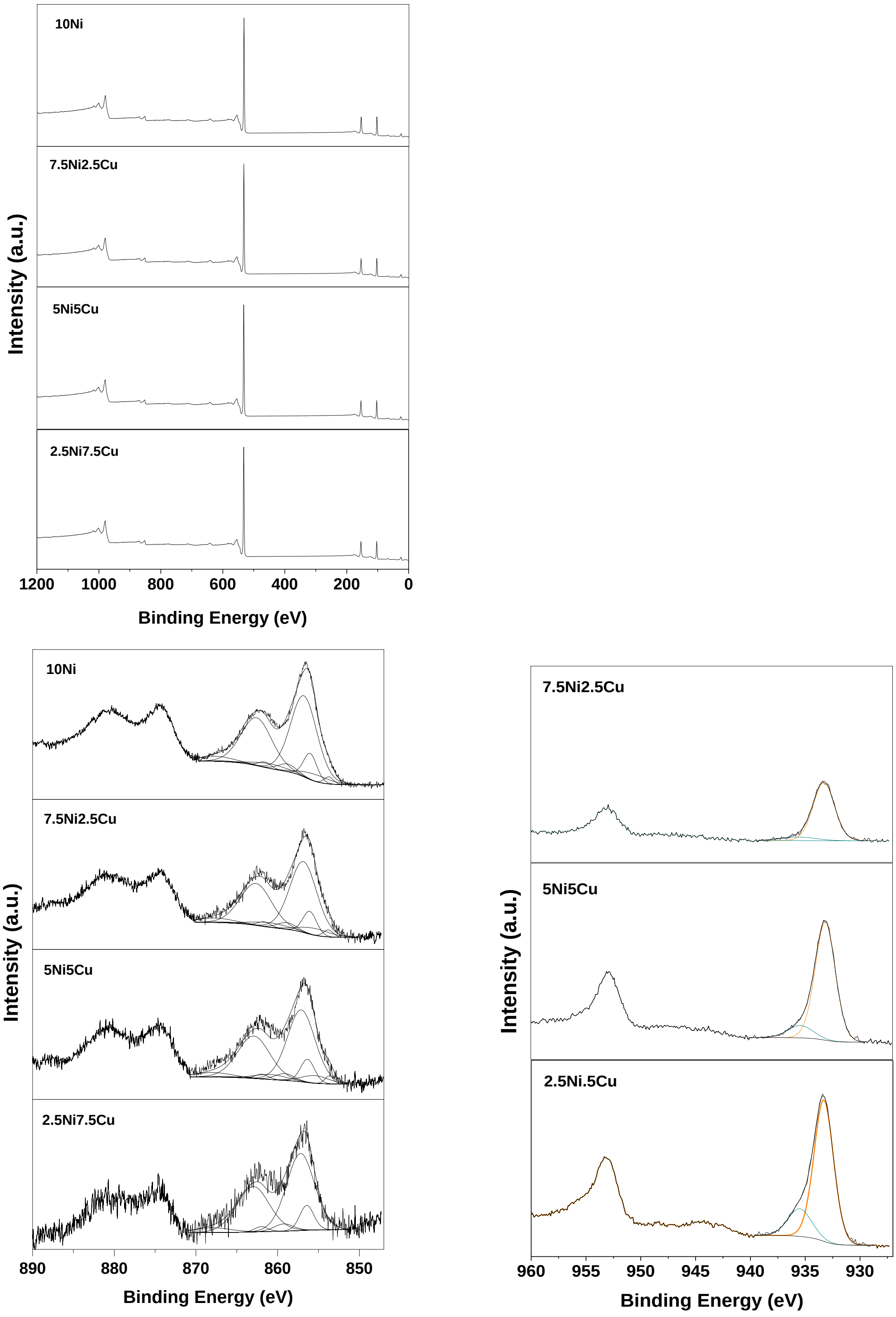

Figure S3. XPS spectrum registered for the calcined samples.

Table S1. Conversion of m-cresol and distribution of products obtained over the NiCu-catalysts at 300 °C and 10 bar.

**Catalyst**	**W/F ($g \cdot h \cdot mol^{-1}$)**	**Conversion (%)**	**Selectivity (%)**				
			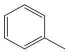	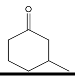	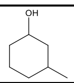	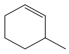	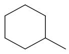
10Ni	1.7	58	11	29	51	2	7
5Ni5Cu	1.7	65	6	28	61	2	3
10Cu	1.7	7	6	26	58	3	7

Table S2. Reaction rates for the transformation of m-cresol and for the main reaction pathways over different supported catalysts, reaction conducted at 300 °C and 10 bar. TOT: total conversion; DDO: direct deoxygenation pathway; HYD: hydrogenation pathway.

Catalyst	r[a] ($mmol\ g_{cat}^{-1} \cdot h^{-1}$)			r[b] ($mmol\ g_{Ni}^{-1} \cdot h^{-1}$)			TOF[c] (h^{-1})
	r_{TOT}	r_{DDO}	r_{HYD}	r_{TOT}	r_{DDO}	r_{HYD}	
10Ni	341	37	304	3481	383	3098	705
5Ni5Cu	381	23	359	7803	468	7335	3271
10Cu	41	2	39	-	-	-	-

[a] constant rate calculated per gram of catalyst and [b] per gram of Ni.

[c] Turnover frequency for m-cresol conversion, considering only Ni as active sites.
